# Multi-step hydration/dehydration mechanisms of rhombohedral Y_2_(SO_4_)_3_: a candidate material for low-temperature thermochemical heat storage†

**DOI:** 10.1039/d0ra02566f

**Published:** 2020-04-21

**Authors:** Kunihiko Shizume, Naoyuki Hatada, Shoko Yasui, Tetsuya Uda

**Affiliations:** Department of Materials Science and Engineering, Kyoto University Yoshida Honmachi, Sakyo-ku Kyoto 606-8501 Japan shizume.kunihiko.26z@st.kyoto-u.ac.jp hatada.naoyuki.8u@kyoto-u.ac.jp uda.tetsuya.5e@kyoto-u.ac.jp

## Abstract

To evaluate rhombohedral Y_2_(SO_4_)_3_ as a new potential material for low-temperature thermochemical energy storage, its thermal behavior, phase changes, and hydration/dehydration reaction mechanisms are investigated. Rhombohedral Y_2_(SO_4_)_3_ exhibits reversible hydration/dehydration below 130 °C with relatively small thermal hysteresis (less than 50 °C). The reactions proceed *via* two reaction steps in approximately 0.02 atm of water vapor pressure, *i.e.* “high-temperature reaction” at 80–130 °C and “low-temperature reaction” at 30–100 °C. The high-temperature reaction proceeds by water insertion into the rhombohedral Y_2_(SO_4_)_3_ host structure to form rhombohedral Y_2_(SO_4_)_3_·*x*H_2_O (*x* = ∼1). For the low-temperature reaction, rhombohedral Y_2_(SO_4_)_3_·*x*H_2_O accommodates additional water molecules (*x* > 1) and is eventually hydrated to Y_2_(SO_4_)_3_·8H_2_O (monoclinic) with changes in the host structure. At a water vapor pressure above 0.08 atm, intermediate Y_2_(SO_4_)_3_·3H_2_O appears. A phase stability diagram of the hydrates is constructed and the potential usage of Y_2_(SO_4_)_3_ for thermal energy upgrades is assessed. The high-temperature reaction may act similarly to an existing candidate, CaSO_4_·0.5H_2_O, in terms of reaction temperature and water vapor pressure. Additionally, the hydration of rhombohedral Y_2_(SO_4_)_3_·*x*H_2_O to Y_2_(SO_4_)_3_·3H_2_O should exhibit a larger heat storage capacity. With respect to the reaction kinetics, the initial dehydration of Y_2_(SO_4_)_3_·8H_2_O to rhombohedral Y_2_(SO_4_)_3_ introduces a microstructure with pores on the micron order, which might enhance the reaction rate.

## Abstract 2

To evaluate rhombohedral Y_2_(SO_4_)_3_ as a new potential material for low-temperature thermochemical energy storage, its thermal behavior, phase changes, and hydration/dehydration reaction mechanisms are investigated.

## Graphical abstract

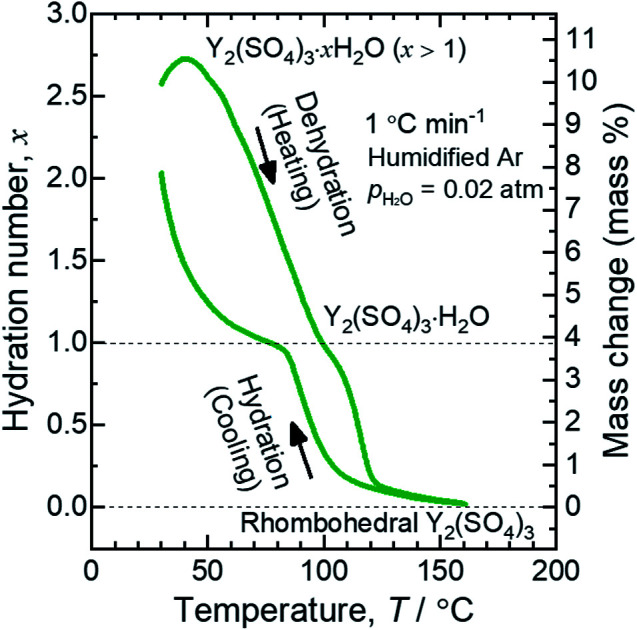

## 1. Introduction

Thermal energy storage based on chemical reactions (TCHS: thermochemical heat storage) is a prospective technology for the reduction of fossil-fuel consumption by storing waste heat.^1,2^ One of the expected roles of TCHS is storing and utilizing heat below 250 °C, where abundant low-grade waste heat and solar energy might be available. For widespread application, a critical challenge is to identify appropriate reversible dehydration/hydration reaction systems satisfying following criteria: suitable reaction temperature, enabling high heat storage capacity, fast enough reaction rate and heat output rate, long-term stability of thermophysical and mechanical properties including, for example, no liquefaction due to melting or deliquescence, non-toxicity of materials themselves and reaction products, the low cost of materials, *etc.*^3^ Solid–gas reactions using water vapor as a reactive gas (dehydration/hydration reactions) are favorable because water is safe and easily stored *via* condensation. To date, various salt hydrates, whose dehydration/hydration reactions can be operated below 250 °C, have been proposed, for example, MgSO_4_·*n*H_2_O (*n* = 0–7),^4–6^ CaCl_2_·*n*H_2_O (*n* = 0–6),^7^ LiOH·H_2_O,^8–10^ Na_2_S·5H_2_O,^11,12^ and CaSO_4_·0.5H_2_O.^13,14^ However, these salt hydrates suffer from some undesirable behaviors related to hydration kinetics and cycle performance.‡‡The major problems of these salt hydrates are described below. MgSO_4_·*n*H_2_O (*n* = 0–7): shape instability due to the melting of the salt which occurs during dehydration of MgSO_4_·7H_2_O to MgSO_4_·6H_2_O.^5^ In addition, the fine powder rapidly forms a skin of the hydrated salt on the surface when it reacts with humid air, which decreases the reaction rate.^6^ CaCl_2_·*n*H_2_O (*n* = 0–6): agglomeration of the salt due to partial melting of the salt during the dehydration or a partial formation of a solution of the salt attributed to the hygroscopicity during rehydration, which decreases the reaction rate.^7^ LiOH·H_2_O: the low hydration reaction rate of pure LiOH which limits the performance of low-temperature TCHS systems.^9,10^ Na_2_S·5H_2_O: generation of toxic by-product H_2_S gas by contacting with water.^12^ CaSO_4_·0.5H_2_O: degradation due to phase transformation from CaSO_4_ (iii) to the low-hydration-reactive phase CaSO_4_ (ii) during repetitive dehydration/hydration reaction.^13,14^ Despite numerous efforts related to the modification of material properties such as developing composite materials based on these salt hydrates, no materials have been widely used yet.

Recently, we reported the dehydration/hydration of β-La_2_(SO_4_)_3_·H_2_O as a new candidate reaction for TCHS below 250 °C.^15–17^ The reactions proceed by insertion/deinsertion of the H_2_O guest molecules into β-La_2_(SO_4_)_3_ host lattices,§§CaSO_4_·0.5H_2_O is also known to dehydrate and rehydrate by deinsertion and insertion of H_2_O guest molecules into the sulfate host lattice.^18^ which leads the rapid and reversible dehydration/hydration reaction even at low-temperature below 250 °C. Furthermore, β-La_2_(SO_4_)_3_ is non-toxic and does not show melting and deliquescence. However, the heat storage capacity is insufficient due to the small changes in the hydration water content (β-La_2_(SO_4_)_3_·H_2_O: 3.2 mass% anhydrous basis). There is still a need to find other materials.

In this study, we focused on Y_2_(SO_4_)_3_ as a new candidate material for the low-temperature TCHS system. Y_2_(SO_4_)_3_ is another trivalent-rare-earth sulfate with a chemical formula analogous to that of β-La_2_(SO_4_)_3_ but it has a crystal structure belonging to the rhombohedral system (space group: No. 148).^19^ Due to the differences of rare-earth elements and crystal structures, the hydration/dehydration behavior of rhombohedral Y_2_(SO_4_)_3_ should differ from that of β-La_2_(SO_4_)_3_ (monoclinic system, space group: 15). Thus it is worthwhile to investigate if rhombohedral Y_2_(SO_4_)_3_ shows favorable hydration/dehydration behaviors. See ESI-1† for their crystal structures. Though there are a few studies associated with the phase changes, reaction temperature, and enthalpy changes on dehydration of Y_2_(SO_4_)_3_·8H_2_O,^20–22^ the backward hydration reaction behaviors have not been reported. In addition, Y_2_O_3,_ the raw material of Y_2_(SO_4_)_3_, is oversupplied, so the cost is not expensive at this time, 3 USD kg^−1^ in 2018.^23^ Therefore, Y_2_(SO_4_)_3_ might be a low-cost candidate for TCHS.

In this work, thermogravimetry (TG) studies of the hydration/dehydration reaction were conducted. High-temperature X-ray diffraction (XRD) investigated the crystal structure changes in the dehydration reaction. Then, we systematically investigated the phases formed in the hydration process under various conditions of temperatures and water vapor pressure *p*_H_2_O_ and discussed the available reactions for TCHS system. In addition, we examined the microstructures of Y_2_(SO_4_)_3_ and Y_2_(SO_4_)_3_·8H_2_O by nitrogen gas adsorption (N_2_ adsorption), optical microscopy, and transmission electron microscopy (TEM) because the microstructures should influence the dehydration/hydration reaction rates.^17^

## 2. Experimental

### 2.1 Materials

In this paper, a-few-hundreds-μm-large particles of Y_2_(SO_4_)_3_·8H_2_O (Strem Chemical, 99.9%) were used as the starting material for the syntheses of rhombohedral Y_2_(SO_4_)_3_ by heating Y_2_(SO_4_)_3_·8H_2_O. The heating processes were either conducted at the time of thermogravimetry, high-temperature XRD and N_2_ adsorption measurements in the respective apparatus or pre-conducted in a stand-alone furnace.

### 2.2 Thermogravimetry (TG)

To investigate the dehydration/hydration reaction behavior of rhombohedral Y_2_(SO_4_)_3_, TG was conducted using a Rigaku Thermo plus TG 8120. Y_2_(SO_4_)_3_·8H_2_O was heated to about 400 °C, which is the temperature where dehydration to the anhydrate should be completed, and subsequently cooled to about 30 °C in the TG apparatus (“first heating–cooling cycle”) under a humidified argon gas (water vapor pressure *p*_H_2_O_ = 0.02 atm) flow. At this point, Y_2_(SO_4_)_3_·*x*H_2_O was obtained as a rehydrated phase of anhydrate Y_2_(SO_4_)_3_. On the subsequent “second heating–cooling cycle” between 30–160 °C, the dehydration/hydration reaction behaviors of Y_2_(SO_4_)_3_·*x*H_2_O were investigated. Isothermal TG measurements at various temperatures in humidified argon (*p*_H_2_O_ = 0.023 atm) were conducted on the hydration of rhombohedral Y_2_(SO_4_)_3_. For isothermal TG measurements, Y_2_(SO_4_)_3_·8H_2_O was used as the starting material and it was dehydrated to rhombohedral Y_2_(SO_4_)_3_ upon heating to 320 °C in dry argon by TG. Subsequently, it was cooled to arbitrary temperatures and the isothermal hydration reaction started by switching flowing gas from dry argon to humidified argon.

In all the TG measurements, the sample material was put in a platinum cylindrical crucible with a diameter of 5 mm and a height of 5 mm and the same empty crucible was used as the reference.

### 2.3 X-ray diffraction (XRD)

XRD measurements were conducted on a PANalytical X'Pert-Pro MPD using Cu Kα radiation. High-temperature data collection was achieved using an Anton Paar HTK1200N high-temperature oven-chamber under humidified oxygen. For the high-temperature XRD experiments, Y_2_(SO_4_)_3_·8H_2_O (Strem Chemical, 99.9%) was heated to 300 °C to form rhombohedral Y_2_(SO_4_)_3_ and subsequently, it was cooled to 50 °C to form the hydrated phase Y_2_(SO_4_)_3_·*x*H_2_O. The XRD patterns during dehydration of Y_2_(SO_4_)_3_·*x*H_2_O were collected on the second heating process from 50 °C to 150 °C. All heating/cooling processes for *in situ* XRD measurements were conducted under 0.03 atm of *p*_H_2_O_. Rietveld refinement analyses were performed with the help of a commercial software X'Pert HighScorePlus (PANalytical) on the *in situ* XRD patterns to identify the phase and the lattice parameters of the samples.

### 2.4 Isothermal hydration treatments of Y_2_(SO_4_)_3_ under various temperature and *p*_H_2_O_

The phases formed by the hydration of Y_2_(SO_4_)_3_ under arbitrary temperatures (37–84 °C) and *p*_H_2_O_ (0.03–0.4 atm) were investigated by isothermal hydration treatments using two electric furnaces. Rhombohedral Y_2_(SO_4_)_3_ was prepared by dehydration of Y_2_(SO_4_)_3_·8H_2_O at 200 °C for 1 hour in air. Rhombohedral Y_2_(SO_4_)_3_ (0.1 g) was placed in an approximately 60 mL closed vessel. The vessel and a tank with water were put in separate furnaces and they were connected by the gas line across the furnaces. The vessel was maintained at a constant temperature for 3 hours while the *p*_H_2_O_ control was performed by flowing humidified argon gas at a rate of 200 mL min^−1^ bubbled through water kept at a constant temperature. A ribbon heater was wrapped around the gas line outside the furnaces and kept over 80 °C to prevent condensation of water vapor in humidified argon gas.

The phases of the sample after the hydration treatments were identified by XRD. Fourteen batches under different conditions of temperature and *p*_H_2_O_ were examined. Based on the results of the isothermal TG and hydration treatments, the phase stability diagram of Y_2_(SO_4_)_3_ with temperature and water vapor pressure axes (*T*–*p*_H_2_O_ map) was constructed.

### 2.5 Nitrogen gas adsorption measurements (N_2_ adsorption)

The specific surface areas of the samples were evaluated by N_2_ adsorption measurements at the liquid nitrogen temperature (77 K) using a BEL JAPAN BELSORP-max. Y_2_(SO_4_)_3_·8H_2_O (5.758 g) and rhombohedral Y_2_(SO_4_)_3_ (1.321 g), which was prepared by heating Y_2_(SO_4_)_3_·8H_2_O at 200 °C for 1 hour in air, were placed in a Pyrex glass tube and subjected to a vacuum heating pretreatment. Afterward, N_2_ adsorption measurements were conducted.

### 2.6 Optical microscope and TEM observations

Change in the morphology of the particles during the dehydration process of Y_2_(SO_4_)_3_·8H_2_O to rhombohedral Y_2_(SO_4_)_3_ was analyzed by optical microscopy using a Nikon L150 L-150II-TI-DIC-A. Y_2_(SO_4_)_3_·8H_2_O was observed by the optical microscope as it is. Rhombohedral Y_2_(SO_4_)_3_ made by heating Y_2_(SO_4_)_3_·8H_2_O at 270 °C for 2 hours in air and was observed by the optical microscope.

The microstructure inside the rhombohedral Y_2_(SO_4_)_3_ particle was observed by TEM using a JEOL JEM-2100F. The rhombohedral Y_2_(SO_4_)_3_ specimen for the TEM observation was cut out from the particle by a focused ion beam (FIB) using a JEOL JIB-4600F.

## 3. Results

### 3.1 Hydration/dehydration reaction behaviors of rhombohedral Y_2_(SO_4_)_3_

**Fig. 1:**
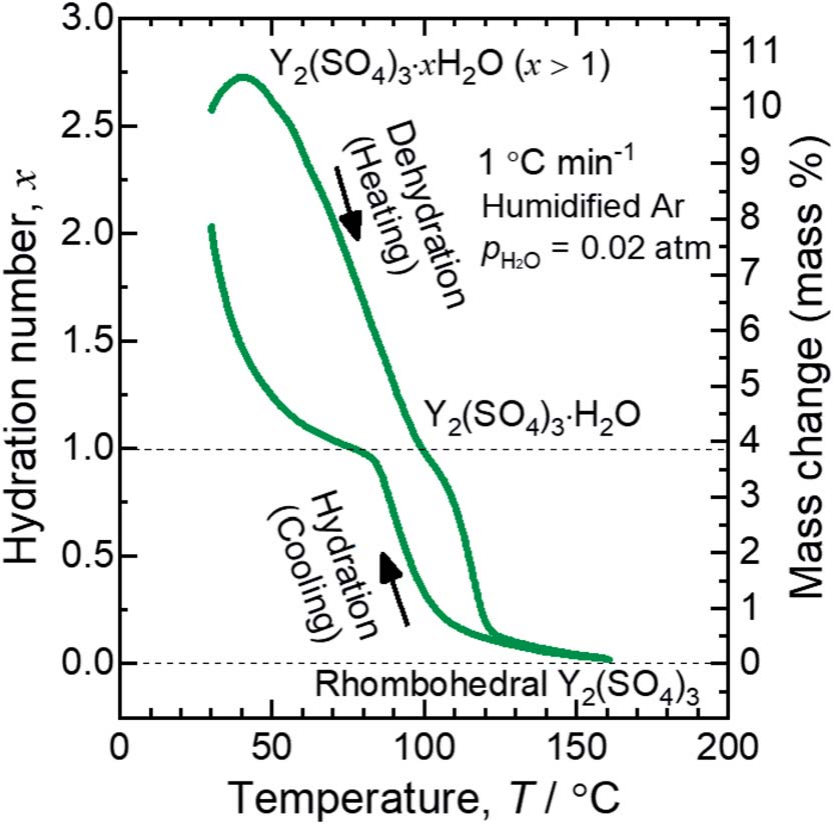
TG curve of rhombohedral Y_2_(SO_4_)_3_ undergoing hydration/dehydration reactions upon cooling/heating. Vertical axis in right side shows the mass changes of the sample. Vertical axis in left side shows apparent hydration number *x* calculated from the mass change. (Note: This data was presented at IMPRES2019.^24^)

Fig. 1 shows a TG curve of rhombohedral Y_2_(SO_4_)_3_ upon cooling/heating in humidified argon. Rhombohedral Y_2_(SO_4_)_3_ can hydrate/dehydrate below 130 °C with relatively small thermal hysteresis (below 50 °C). The change in the hydration number *x* is greater than 1 and the total mass change in the hydration water content is larger than 10 mass% anhydrous basis. This value is larger than that of La_2_(SO_4_)_3_ (3 mass%) and CaSO_4_ (6 mass%) and a higher heat storage capacity is expected. Therefore, it may be a suitable material for the temperature range.

The TG curve (Fig. 1) suggests that the dehydration/hydration reactions proceed *via* two reaction steps. The temperature ranges of these two reaction steps are overlapping, preventing them from being clearly distinguished. Between 80–130 °C, apparent hydration number *x* varies steeply from 0 to approximately 1 (“high-temperature dehydration/hydration reaction”). Below 80 °C, Y_2_(SO_4_)_3_·H_2_O forms a hydrate with a larger hydration number in the cooling process. The dehydration/hydration reaction proceeds at 30–100 °C (“low-temperature dehydration/hydration reaction”). Both the high-temperature reaction and the low-temperature reaction may be useful for TCHS system. On the other hand, the phase changes in each reaction step are not established. Rosso *et al.* already reported the relationship between the reaction temperatures and formed hydrate phases of Y_2_(SO_4_)_3_. Y_2_(SO_4_)_3_·4H_2_O is generated as an intermediate phase in the dehydration process of Y_2_(SO_4_)_3_·8H_2_O to Y_2_(SO_4_)_3_.^21^ In contrast, our TG curve (Fig. 1) of rhombohedral Y_2_(SO_4_)_3_ suggest another hydrate phase Y_2_(SO_4_)_3_·H_2_O. Here, we further investigate in detail the high-temperature and the low-temperature dehydration/hydration reactions.

#### 3.1.1 High-temperature dehydration/hydration reaction

**Fig. 2:**
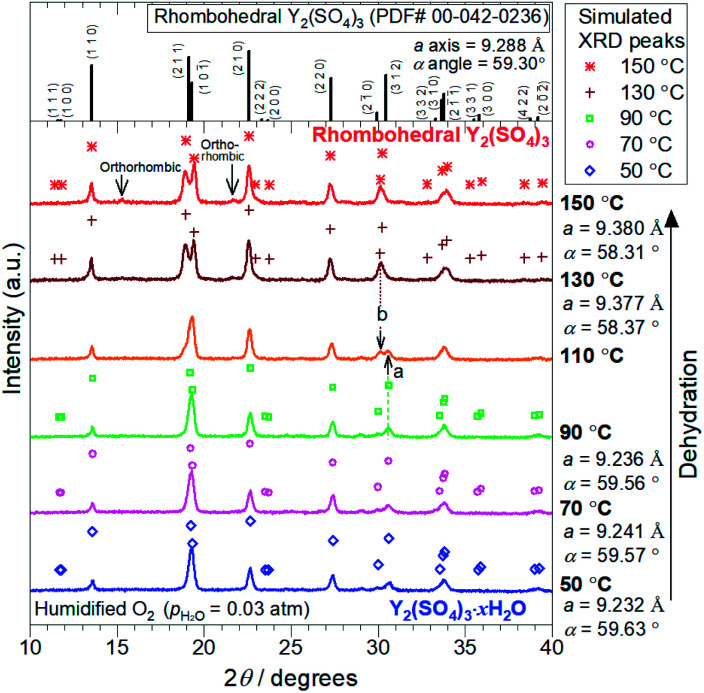
High-temperature XRD patterns collected during the dehydration reaction of Y_2_(SO_4_)_3_·*x*H_2_O to rhombohedral Y_2_(SO_4_)_3_ in the heating process under humidified oxygen (*p*_H_2_O_ = 0.03 atm). Topmost chart shows the reported XRD pattern of rhombohedral Y_2_(SO_4_)_3_, which is cited from ICDD (PDF#00-042-0236). The other part shows the measured XRD patterns and the simulated XRD peaks at each temperature. Marks shown above XRD patterns denote simulated XRD peaks obtained by Rietveld refinement using rhombohedral Y_2_(SO_4_)_3_ (PDF#00-042-0236) as the initial crystal structure. The diffraction angles of simulated XRD peaks are determined by the results of lattice parameter refinements shown on the right side of the figure below each measurement temperature. Note that the intensities of simulated XRD peaks are assumed to be the same as the reported pattern (PDF#00-042-0236). Peak a and peak b in the XRD pattern at 110 °C, indicated by black arrows, are attributed to Y_2_(SO_4_)_3_·*x*H_2_O and rhombohedral Y_2_(SO_4_)_3_ respectively. Two weak peaks, indicated as “orthorhombic”, in XRD pattern at 150 °C represent the slight formation of orthorhombic Y_2_(SO_4_)_3_. The peaks are identified by the reported pattern of orthorhombic Y_2_(SO_4_)_3_ (ICDD, PDF#04-009-9561).

The crystal structure changes from Y_2_(SO_4_)_3_·*x*H_2_O to rhombohedral Y_2_(SO_4_)_3_ during the dehydration reaction were investigated by high-temperature XRD. Fig. 2 shows XRD patterns during dehydration upon heating from 50 °C to 150 °C under humidified oxygen (*p*_H_2_O_ = 0.03 atm) and the reported diffraction peak positions of rhombohedral Y_2_(SO_4_)_3_ (PDF#00-042-0236). Based on TG curves (Fig. 1), the XRD patterns below 110 °C (50, 70, and 90 °C) represent Y_2_(SO_4_)_3_·*x*H_2_O (*x* ≥ 1, depending on temperature), while those at higher temperatures (130 and 150 °C) represent rhombohedral Y_2_(SO_4_)_3_. The marks shown above each XRD pattern except one at 110 °C represent the simulated XRD peaks based on the crystal structure of rhombohedral Y_2_(SO_4_)_3_ with modified lattice parameters (*a* axial length, *α* angle) derived by Rietveld refinement. As can be seen in Fig. 2, the simulated and measured XRD peaks agree well at every temperature. Since the XRD patterns at each temperature are interpreted as rhombohedral Y_2_(SO_4_)_3_ structure with the changed lattice parameters, the hydration reaction of rhombohedral Y_2_(SO_4_)_3_ should proceed by water insertion into the host lattice. In particular, the overlapping patterns of the Y_2_(SO_4_)_3_·*x*H_2_O and rhombohedral Y_2_(SO_4_)_3_ observed only at 110 °C, represented by the co-existing of “peak a” and “peak b” derived from the hydrate and anhydrate phases respectively. This should account for the steep high-temperature dehydration reaction of Y_2_(SO_4_)_3_·*x*H_2_O to rhombohedral Y_2_(SO_4_)_3_.

Hereinafter we refer to the low-temperature hydrate phase below 110 °C as “rhombohedral Y_2_(SO_4_)_3_·*x*H_2_O (*x* ≥ 1)” and the reaction formula of the high-temperature reaction is written as follows:Rhombohedral Y_2_(SO_4_)_3_ (s) + H_2_O (g) ⇄ Rhombohedral Y_2_(SO_4_)_3_·H_2_O (s)

At the lower temperatures (between 50–90 °C), the XRD patterns do not exhibit marked changes, but the hydration number *x* changes with temperature (see Fig. 1), suggesting that rhombohedral Y_2_(SO_4_)_3_·*x*H_2_O (*x* ≥ 1) has a nonstoichiometric hydration number.

#### 3.1.2 Low-temperature dehydration/hydration reaction

**Fig. 3:**
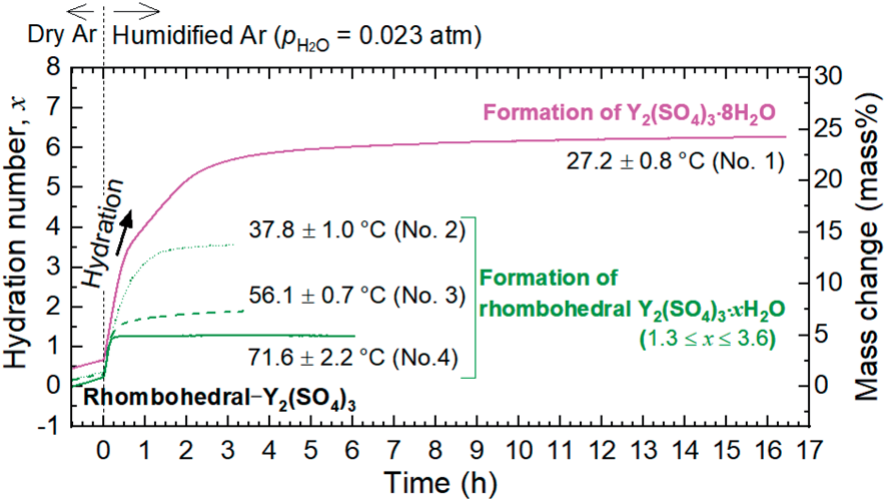
TG curves of rhombohedral Y_2_(SO_4_)_3_ of the isothermal hydration reaction at four different temperatures: 27.2 °C (No. 1), 37.8 °C (No. 2), 56.1 °C (No. 3), and 71.6 °C (No. 4). Temperature during the measurements fluctuated up to 2.2 °C. For these measurements, Y_2_(SO_4_)_3_·8H_2_O was used as the starting material. It was dehydrated to rhombohedral Y_2_(SO_4_)_3_ at 320 °C in TG under dry argon. Then it was cooled to a predetermined temperature. The sample mass of rhombohedral Y_2_(SO_4_)_3_ at around 320 °C, used for the basis of mass change, are as follows: 14.17 mg (No. 1), 14.34 mg (No. 2), 13.75 mg (No. 3), 13.54 mg (No. 4). While maintaining a constant temperature, the isothermal hydration reaction started by switching the flowing gas from dry argon to humidified argon (*p*_H_2_O_ = 0.023 atm) to examine the hydration process. Horizontal axis shows the elapsed time since the initiation of the hydration reaction by switching the flowing gas from dry argon to humidified argon.

**Fig. 4:**
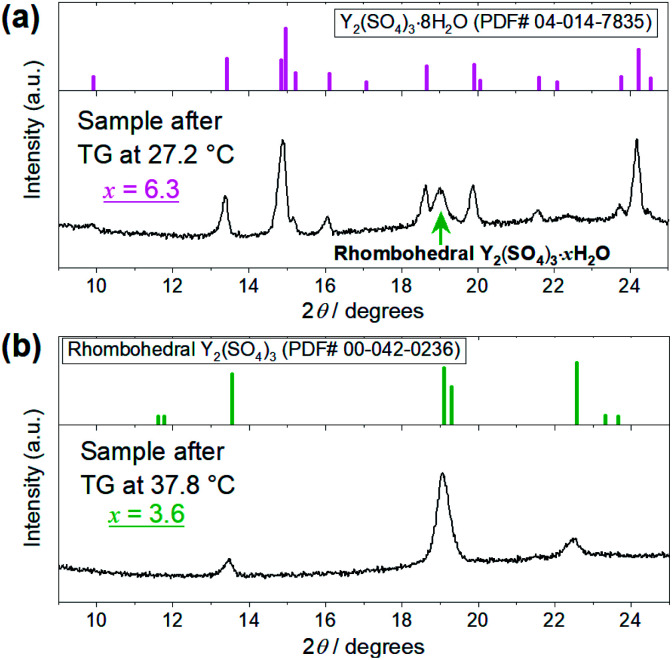
XRD patterns of the Y_2_(SO_4_)_3_·*n*H_2_O samples after the TG measurements at (a) 27.2 °C and (b) 37.8 °C shown in Fig. 3. (a) Top figure represents the reference pattern of Y_2_(SO_4_)_3_·8H_2_O (ICDD (PDF#04-014-7835)). In the bottom figure, there is an XRD peak attributed to the residual rhombohedral Y_2_(SO_4_)_3_·*x*H_2_O around 19°. (b) Top figure represents the reference pattern of rhombohedral Y_2_(SO_4_)_3_ (ICDD (PDF#00-042-0236)).

**Fig. 5:**
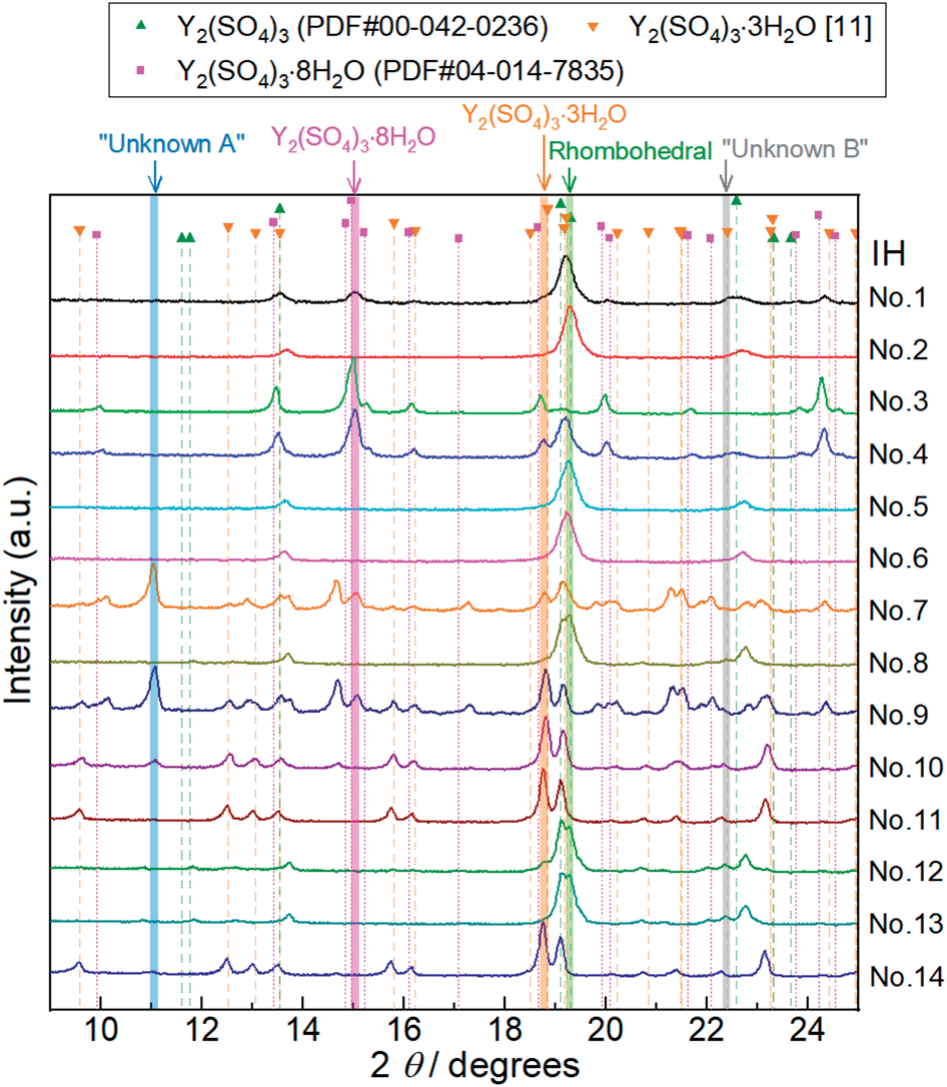
XRD patterns of Y_2_(SO_4_)_3_ hydrate samples after 14 batches of isothermal hydration treatments (IH No. 1–14). Characteristic diffraction angles where distinctive peaks of Y_2_(SO_4_)_3_·8H_2_O, Y_2_(SO_4_)_3_·3H_2_O,^25^ rhombohedral Y_2_(SO_4_)_3_·*x*H_2_O (Rhombohedral), Unknown A, and Unknown B exist are highlighted by pink, orange, green, blue, and gray, respectively. Peaks at 11.1° highlighted by a blue belt (No. 7, 9, 10, and 14) do not match any of the reported reference pattern peaks. These peaks are attributed to an unknown hydrated phase Unknown A. Similarly, we assumed that the peak at 22.4° highlighted by gray (No. 8, 12, and 13) is attributed to Unknown B. Doublet peaks around 19° are also attributed to Unknown B.

To elucidate the phase changes and the reaction behaviors in the low-temperature hydration reaction, we conducted isothermal TG measurements and isothermal hydration treatments (IH). Table 1 shows the hydration conditions, formed phases, and the largest hydration numbers reached in each isothermal TG measurement and isothermal hydration treatment (IH No. 1–14). Fig. 3 shows the isothermal TG curves obtained by the above measurements. Fig. 4 and 5 show the XRD patterns of the samples after the isothermal TG measurements and isothermal hydration treatments. The results in Fig. 3, 4 and 5 are summarized in Table 1.

**Table 1:** Summary of the results of the isothermal TG measurements and the isothermal hydration (IH) treatments

Expt. #	Temp. (°C)	*p*_H_2_O_ (atm)	Time (h)	*x* b	Detected hydrated phases^c^				
					Y_2_(SO_4_)_3_·8H_2_O	Y_2_(SO_4_)_3_·3H_2_O	Rhombo-hedral^d^	Unknown A	Unknown B
TG 1	27.2	0.023	3^a^	6.3	Strong	—	Weak	—	—
TG 2	37.8	0.023	1^a^	3.6	Weak	—	Strong	—	—
TG 3	56.1	0.023	1^a^	1.9	Not measured by XRD				
TG 4	71.6	0.023	0.2^a^	1.3	Not measured by XRD				
IH 1	38	0.043	3	—	Weak	—	Strong	—	—
IH 2	37	0.030	3	—	—	—	Strong	—	—
IH 3	55	0.13	3	—	Strong	Weak	—	—	—
IH 4	56	0.10	4	—	Strong	Weak	—	—	—
IH 5	55	0.088	3	—	—	—	Strong	—	—
IH 6	55	0.079	3	—	—	—	Strong	—	—
IH 7	69	0.24	3	—	Weak	Weak	—	Strong	—
IH 8	69	0.14	3	—	—	—	Strong	—	Weak
IH 9	79	0.40	4	—	Weak	Weak	—	Strong	—
IH 10	79	0.36	3	—	—	Strong	—	Weak	—
IH 11	79	0.32	3	—	—	Strong	—	—	—
IH 12	78	0.18	3	—	—	Weak	Strong	—	Weak
IH 13	78	0.14	3	—	—	—	Strong	—	Weak
IH 14	84	0.44	3	—	—	Strong	—	Weak	—

a “Time” for TG measurements represents the time required for a 90% mass increase relative to the final achieved mass at the end of the measurement.

b *x*: hydration number.

c Detected hydrated phases: hydrated phases detected by XRD measurements in the samples after the isothermal TG measurements and the isothermal hydration treatments (No. 1–14). “Strong” and “weak” represent the relative intensity of the XRD peaks derived from each phase. The phase with “strong” corresponds to the main phase formed in the hydration treatments (TG 1–4, IH 1–14) whereas the phase with “weak” corresponds to the secondary phases.

d Rhombohedral: Rhombohedral Y_2_(SO_4_)_3_·*x*H_2_O

Depending on the hydration conditions, one or more of three known phases Y_2_(SO_4_)_3_·8H_2_O (monoclinic), Y_2_(SO_4_)_3_·3H_2_O (orthorhombic),^25^^,^¶¶The crystal structure of Y_2_(SO_4_)_3_·3H_2_O is considered to be the same as that of Yb_2_(SO_4_)_3_·3H_2_O reported by Mills *et al.*^25^ The XRD pattern of Y_2_(SO_4_)_3_·3H_2_O shown in Fig. 5 as orange inverted triangles was estimated by adjusting only the diffraction angles of XRD peaks of Yb_2_(SO_4_)_3_·3H_2_O while the peak intensities were left as is. and rhombohedral Y_2_(SO_4_)_3_·*x*H_2_O as well as two unknown phases (“Unknown A” and “Unknown B”) are present. Based on the isothermal TG results in Fig. 3, rhombohedral Y_2_(SO_4_)_3_·H_2_O, which is formed by the high-temperature hydration reaction, further is hydrated to rhombohedral Y_2_(SO_4_)_3_·*x*H_2_O (*x* > 1) and becomes Y_2_(SO_4_)_3_·8H_2_O (monoclinic) at lower temperatures. Isothermal hydration treatments under relatively high *p*_H_2_O_ (>0.08 atm) reveal that another intermediate hydrate Y_2_(SO_4_)_3_·3H_2_O appears at intermediate temperature.

The isothermal TG measurements indicate that rhombohedral Y_2_(SO_4_)_3_·*x*H_2_O has a nonstoichiometric hydration number *x* (>1). Fig. 3 shows the isothermal TG curves (TG No. 1–4) in the hydration reaction of rhombohedral Y_2_(SO_4_)_3_ at a constant temperature of 27.2 (No. 1), 37.8 (No. 2), 56.1 (No. 3), and 71.6 °C (No. 4) under 0.023 atm of *p*_H_2_O_. The apparent increase in the hydration number *x* becomes larger as the holding temperature is lowered: *x* = 1.3 (at 71.6 °C), 1.9 (at 56.1 °C), 3.6 (at 37.8 °C), and 6.3 (at 27.2 °C). Fig. 4(a) and (b) show the XRD patterns of the samples after the isothermal TG measurements at 27.2 °C and 37.8 °C. The pattern of the 6.3 hydrate-equivalent sample confirms the partial formation of Y_2_(SO_4_)_3_·8H_2_O (monoclinic), while the pattern of the 3.6 hydrate-equivalent sample indicates a single-phasic pattern quite similar to that of rhombohedral Y_2_(SO_4_)_3_·*x*H_2_O shown in Fig. 2. Therefore, rhombohedral Y_2_(SO_4_)_3_·*x*H_2_O may have a nonstoichiometric hydration number *x* and accommodate water molecules form *x* = 1 to at least *x* = 3.6. Such non-stoichiometry of hydrates has also been reported for MgSO_4_ – H_2_O system.^4^

In the 6.3 hydrate-equivalent sample, the rhombohedral Y_2_(SO_4_)_3_·*x*H_2_O phase still remains even after the isothermal TG curve (No. 1) reaches a plateau. This suggests that the hydration reaction of rhombohedral Y_2_(SO_4_)_3_·*x*H_2_O to Y_2_(SO_4_)_3_·8H_2_O was significantly slowed down in the middle of the reaction but the cause is unclear at this time. Based on Gibbs' phase rule, the equilibrium solid phase in the hydration reaction should be a single-phase. Thus Y_2_(SO_4_)_3_·8H_2_O is regarded as the equilibrium phase in the conditions (27.2 °C, *p*_H_2_O_ = 0.023 atm).||||Focusing only on condensed phases in the Y_2_(SO_4_)_3_–H_2_O pseudo-binary system, the degree of freedom *F* is represented as follows: *F* = *C* − *P* + 2 = 4 − *P*, where *C* is the number of independent components and *P* is the number of condensed phases. In the case of the isothermal hydration reaction of Y_2_(SO_4_)_3_, the temperature and the total pressure are fixed and the chemical potential of H_2_O is determined by the water vapor pressure of gas phase equilibrated with the Y_2_(SO_4_)_3_–H_2_O system. Then the degree of freedom becomes *F* = 1 − *P*. Because the *F* should be larger than zero, the number of the solid phase should be 1 at most.

**Fig. 6:**
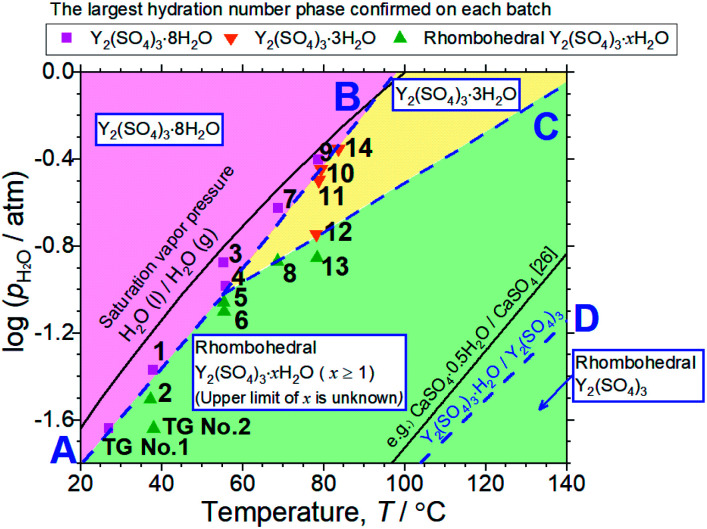
*T*–*p*_H_2_O_ map derived from 14 batches of hydration treatments that expose rhombohedral Y_2_(SO_4_)_3_ under various temperatures and *p*_H_2_O_ for 3 hours. Here, only batches No. 4 and 9 are held for 4 hours. Additionally, the results of the isothermal TG measurements (Fig. 3) and the XRD (Fig. 4) are also represented by “TG No. 1” and “TG No. 2”. Pink squares represent batches where Y_2_(SO_4_)_3_·8H_2_O is formed. Orange inverted triangles represent batches where Y_2_(SO_4_)_3_·3H_2_O is formed but Y_2_(SO_4_)_3_·8H_2_O is not. Green triangles represent batches where neither Y_2_(SO_4_)_3_·3H_2_O and Y_2_(SO_4_)_3_·8H_2_O are formed. Two-phase equilibria are represented by broken lines. Broken lines A and B show the minimum *p*_H_2_O_ and maximum temperature required to form Y_2_(SO_4_)_3_·8H_2_O. Broken line C also shows those required to form Y_2_(SO_4_)_3_·3H_2_O. Broken line D represents the equilibrium between rhombohedral Y_2_(SO_4_)_3_ and rhombohedral Y_2_(SO_4_)_3_·H_2_O. It is drawn using the estimated enthalpy and entropy changes of the dehydration/hydration reaction, determined by TG measurements under four different *p*_H_2_O_. See ESI-2† for the TG curves. Temperature dependences of the saturation vapor pressure of liquid water and the equilibrium between CaSO_4_ and CaSO_4_·0.5H_2_O are also shown.^26^

According to the isothermal TG curves, the hydration reactions are almost complete in 3 hours. Therefore, it is reasonable that the equilibrium phases are contained in the detected phases in each batch (No. 1–14) and we can assume that the phase with the largest hydration number is the equilibrium hydrate for each condition. Based on this assumption, we constructed the phase stability diagram of Y_2_(SO_4_)_3_ hydrates with temperature and water vapor pressure (*T*–*p*_H_2_O_ map) shown in Fig. 6. Only Y_2_(SO_4_)_3_·8H_2_O (monoclinic), Y_2_(SO_4_)_3_·3H_2_O (orthorhombic), and rhombohedral Y_2_(SO_4_)_3_·*x*H_2_O are considered.

Both Unknown A and Unknown B are not considered in constructing the *T*–*p*_H_2_O_ map for the following reasons. Unknown A and Unknown B are some hydrate phases of Y_2_(SO_4_)_3_. Based on the detected conditions for them, the estimated hydration number of Unknown A is between 3 to 8 and that of Unknown B is between 1 to 3. Unknown A and Unknown B might have stable *T*–*p*_H_2_O_ regions near the broken lines B and C respectively but the regions should not be wide because they are always accompanied by rhombohedral Y_2_(SO_4_)_3_·*x*H_2_O, Y_2_(SO_4_)_3_·3H_2_O, or Y_2_(SO_4_)_3_·8H_2_O (see Table 1). As Unknown A and Unknown B are intermediate hydrate phases between the considered phases, even though they are omitted in Fig. 6, it should be sufficient to know the outline of the relationship between the hydration conditions and hydration number changes.

### 3.2 Microstructures of rhombohedral Y_2_(SO_4_)_3_

**Fig. 7:**
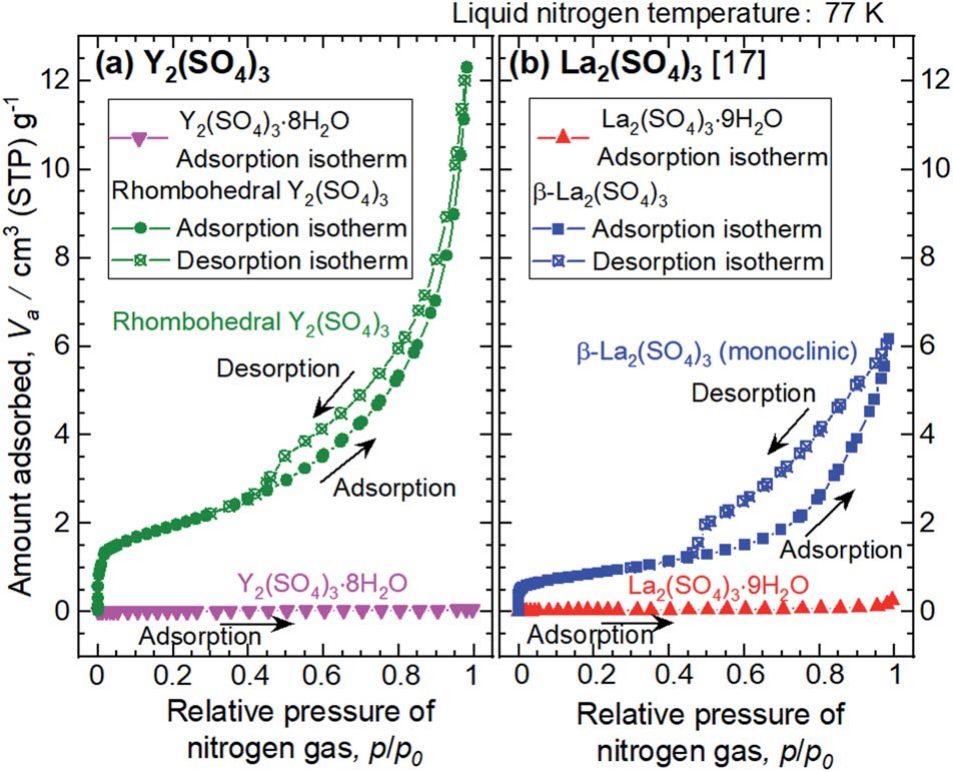
Adsorption/desorption isotherms obtained by N_2_ adsorption measurements on (a) Y_2_(SO_4_)_3_ and (b) La_2_(SO_4_)_3_ for comparison. Pink inverted triangles show the isotherms of Y_2_(SO_4_)_3_·8H_2_O. Green circles denote the isotherms of rhombohedral Y_2_(SO_4_)_3_ prepared by heating Y_2_(SO_4_)_3_·8H_2_O at 200 °C for 1 hour in air. Both samples are measured after a vacuum pretreatment for 1000 minutes at room temperature. Phase of both samples unchanged after N_2_ adsorption measurements, which is confirmed by XRD. Vertical axis represents *V*_a_, which means the volume of adsorbed nitrogen gas under standard temperature and pressure (STP). Here, the temperature is 273.15 K and the gas pressure is 101.325 kPa. Horizontal axis shows the relative pressure *p*/*p*_0_ where *p* is the equilibrium pressure and *p*_0_ is the saturation nitrogen gas pressure over liquid nitrogen at 77 K.

**Fig. 8:**
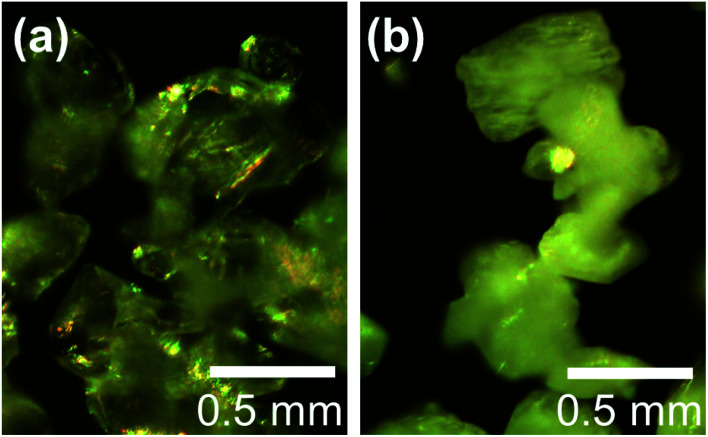
Optical microscope images of (a) Y_2_(SO_4_)_3_·8H_2_O and (b) rhombohedral Y_2_(SO_4_)_3_·*x*H_2_O particles. Rhombohedral Y_2_(SO_4_)_3_ made from Y_2_(SO_4_)_3_·8H_2_O by heating at 270 °C for 2 hour in air. Both particles look yellowish due to the light source of the optical microscope. Actually, Y_2_(SO_4_)_3_·8H_2_O is colorless and rhombohedral Y_2_(SO_4_)_3_ is white under white light.

**Fig. 9:**
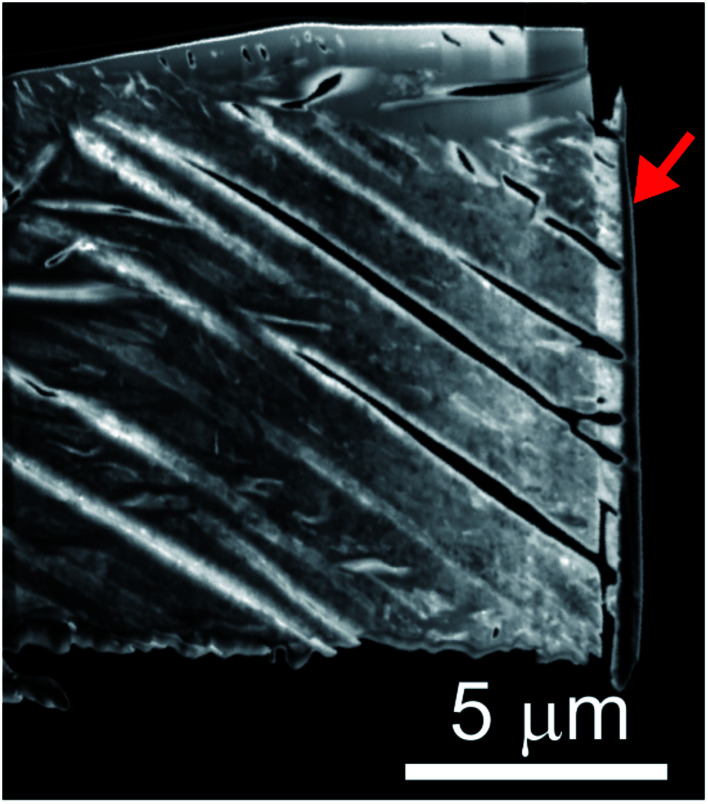
Dark-field TEM image of the rhombohedral Y_2_(SO_4_)_3_ specimen cut out from the rhombohedral Y_2_(SO_4_)_3_ particle by FIB processing. Rhombohedral Y_2_(SO_4_)_3_ made from Y_2_(SO_4_)_3_·8H_2_O by heating at 200 °C for 1 hour in air. Vertical black few-hundred-nm-thick layer at the right edge of the sample indicated by the red arrow is deposited carbon for FIB processing following the same procedure used in ref. 17.

The microstructures of rhombohedral Y_2_(SO_4_)_3_ obtained by dehydration of Y_2_(SO_4_)_3_·8H_2_O were investigated. Fig. 7(a) shows the N_2_ adsorption isotherms of Y_2_(SO_4_)_3_·8H_2_O and rhombohedral Y_2_(SO_4_)_3_. The specific surface area of rhombohedral Y_2_(SO_4_)_3_ by the BET method (7 m^2^ g^−1^) is more than a hundredfold larger than that of the Y_2_(SO_4_)_3_·8H_2_O (0.05 m^2^ g^−1^). ESI-3† shows the BET plots. Fig. 8 shows photographs of (a) Y_2_(SO_4_)_3_·8H_2_O and (b) rhombohedral Y_2_(SO_4_)_3_·*x*H_2_O particles observed by optical microscopy. Note that the hydration number *x* of the rhombohedral phase was not known unclear accurately because rhombohedral Y_2_(SO_4_)_3_ hydrates to some extent in air. Y_2_(SO_4_)_3_·8H_2_O particles show facet planes and high transparency, and they are likely a-few-hundreds-μm-large single crystals. On the other hand, rhombohedral Y_2_(SO_4_)_3_·*x*H_2_O particles are less transparent representing the polycrystalline nature. Correspondingly, a dark-field TEM image of rhombohedral Y_2_(SO_4_)_3_ in Fig. 9 reveals crevices and voids at a-few-micrometer intervals.**We did not conduct TEM observations on Y_2_(SO_4_)_3_·8H_2_O due to the difficulty with sample preparation. The dehydration reaction of Y_2_(SO_4_)_3_·8H_2_O proceeded even under a vacuum at room temperature on FIB processing for sample preparation. A similar microstructure formation is observed in the case of dehydration of La_2_(SO_4_)_3_·9H_2_O to β-La_2_(SO_4_)_3_ (Fig. 7(b)), which is known to improve the kinetics of hydration/dehydration reaction of β-La_2_(SO_4_)_3_.^17^ The microstructure of rhombohedral Y_2_(SO_4_)_3_ is also expected to promote the hydration/dehydration reaction rates by extending the surface area and shortening the diffusion length of water in the crystals although it was not confirmed yet.

## 4. Discussion

### 4.1 Relationships between water insertion mechanisms and reaction temperature ranges: comparison of rhombohedral Y_2_(SO_4_)_3_ and β-La_2_(SO_4_)_3_

**Fig. 10:**
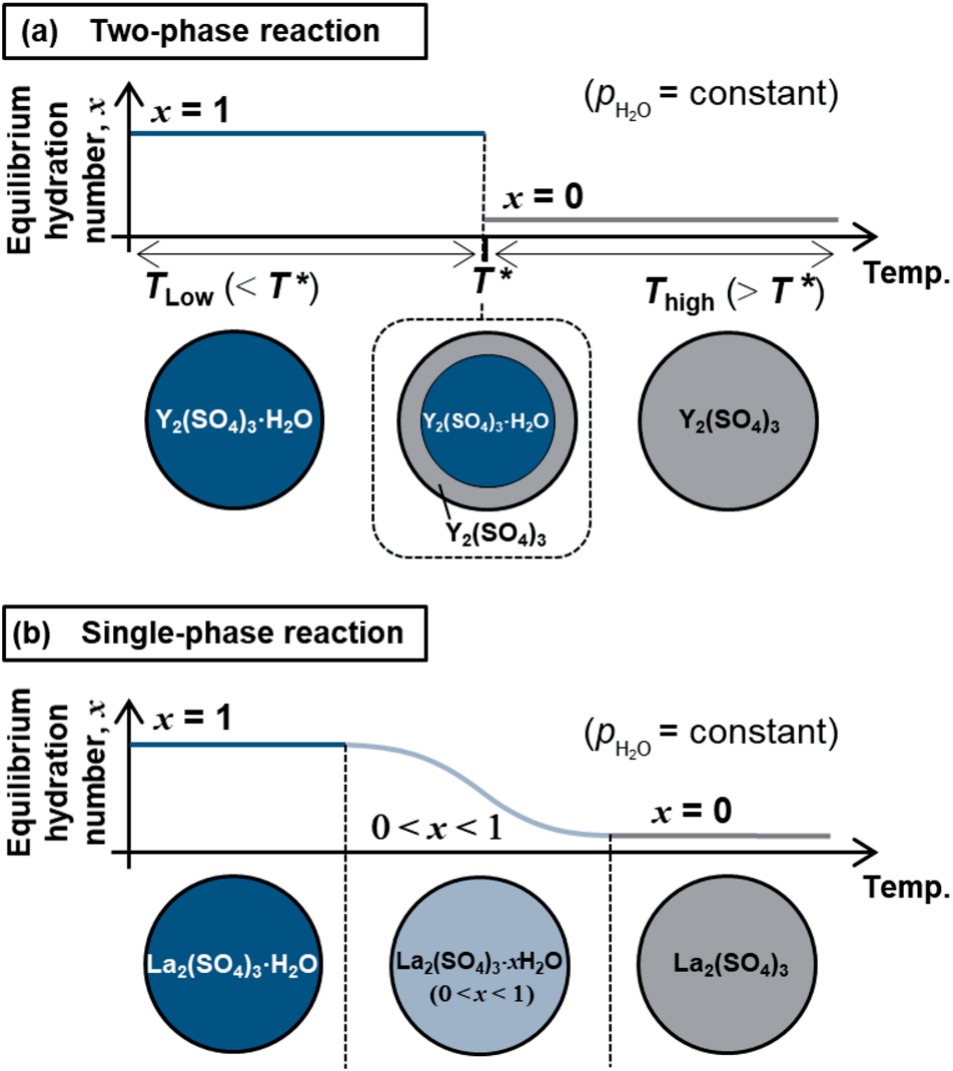
Schematic diagrams of the dehydration processes for (a) the two-phase reaction of rhombohedral Y_2_(SO_4_)_3_ and (b) the single-phase reaction of β-La_2_(SO_4_)_3_. (a) Top figure is the schematic graph showing the change of equilibrium hydration number *x* with temperature increase. Here, T* represents the equilibrium temperature of the two-phase reaction between rhombohedral Y_2_(SO_4_)_3_ and rhombohedral Y_2_(SO_4_)_3_·H_2_O. The schematic diagram under the graph shows the schematic of hydration number distribution of rhombohedral Y_2_(SO_4_)_3_·H_2_O spherical particle during heating. When the temperature is below T*, the equilibrium phase is rhombohedral Y_2_(SO_4_)_3_·H_2_O. At the equilibrium temperature T*, both phases are co-existing until the dehydration reaction finishes. Then rhombohedral Y_2_(SO_4_)_3_ is the equilibrium phase when the temperature is upon T*. (b) The schematic graph showing the continuous change of equilibrium hydration number of β-La_2_(SO_4_)_3_·*x*H_2_O with temperature increase. The schematic diagram under the graph shows the schematic of hydration number distribution of β-La_2_(SO_4_)_3_·*x*H_2_O spherical particle during the dehydration reaction based on the single-phase reaction.

The high-temperature hydration reaction of rhombohedral Y_2_(SO_4_)_3_·*x*H_2_O (*x* = 0 or ∼1) likely proceeds by water insertion into the crystal lattice (see Section 3.1.1). This reaction is similar to the hydration reaction of β-La_2_(SO_4_)_3_·*x*H_2_O (0 ≤ *x* ≤ 1) (monoclinic).^15^ However, their water insertion mechanisms differ. Regarding β-La_2_(SO_4_)_3_·*x*H_2_O, the hydration number *x* changes continuously with maintaining a single-phase nature during the dehydration/hydration reactions. In contrast, the high-temperature hydration reaction of rhombohedral Y_2_(SO_4_)_3_ is a “two-phase reaction” because two different phases (rhombohedral Y_2_(SO_4_)_3_ and rhombohedral Y_2_(SO_4_)_3_·H_2_O) co-exist during the dehydration at 110 °C (see Section 3.1.1 and Fig. 2). Fig. 10 schematically depicts the dehydration processes of (a) the two-phase reaction and (b) the “single-phase reaction”. The two-phase reaction proceeds as the ratio of hydrated and dehydrated phases changes, while the single-phase reaction proceeds as the hydration number of the single solid phase continuously decreases. A two-phase reaction proceeds at a single equilibrium temperature in principle. Thus, the two-phase reaction with a sufficiently fast reaction rate can complete reversible reactions in a narrow temperature range (*e.g.*, rhombohedral Y_2_(SO_4_)_3_·*x*H_2_O (*x* = 0 ⇄ ∼1): 80–130 °C at a rate of 1 °C min^−1^). On the other hand, a single-phase reaction advances with changing the hydration number and the equilibrium temperature continuously in principle. Even if the reaction rate is fast enough, the reaction temperature range broadens (*e.g.*, β-La_2_(SO_4_)_3_·*x*H_2_O: 80–250 °C at a rate of 1 °C min^−1^ (ref. 15)). Therefore, the two-phase reaction with a sufficiently fast reaction rate is preferred for the TCHS system as the required temperature range for reaction completion narrows.

### 4.2 Applicability of rhombohedral Y_2_(SO_4_)_3_ as a TCHS material

Based on the *T*–*p*_H_2_O_ map (Fig. 6), we consider an application of several reactions of rhombohedral Y_2_(SO_4_)_3_ to chemical heat pumps to upgrade thermal energy. The application employs an apparatus consisting of a reactor containing rhombohedral Y_2_(SO_4_)_3_ and an evaporator containing water. In the heat releasing step, saturated water vapor is transported from the evaporator to the reactor and rhombohedral Y_2_(SO_4_)_3_ is hydrated. Here, the temperature lift (the temperature difference between the evaporator and the reactor) should ideally be equal to the difference between the dew point of water and the equilibrium temperature of the hydration reaction at a constant *p*_H_2_O_. The equilibrium temperatures, the temperature lifts attained by reactions A, B, C, and D shown in Fig. 6 are represented by the temperature differences between the saturation vapor pressure line and the broken lines A, B, C, and D at a constant *p*_H_2_O_ (Fig. 6). As can be seen, reaction D (high-temperature reaction: Y_2_(SO_4_)_3_/Y_2_(SO_4_)_3_·H_2_O) works similarly to the hydration reaction of CaSO_4_ in terms of the temperature lift. Reaction C (Y_2_(SO_4_)_3_·*x*H_2_O/Y_2_(SO_4_)_3_·3H_2_O) may also be utilized under high *p*_H_2_O_ (> ∼0.08 atm). On the other hand, using the two-step reaction of C and D is disadvantageous in that they output heat at different temperatures which leads to a large temperature drop against stored heat. The reactions A (Y_2_(SO_4_)_3_·*x*H_2_O/Y_2_(SO_4_)_3_·8H_2_O) and B (Y_2_(SO_4_)_3_·3H_2_O/Y_2_(SO_4_)_3_·8H_2_O) may not be suitable for practical applications due to the small temperature lift (approximately 10 °C).

**Fig. 11:**
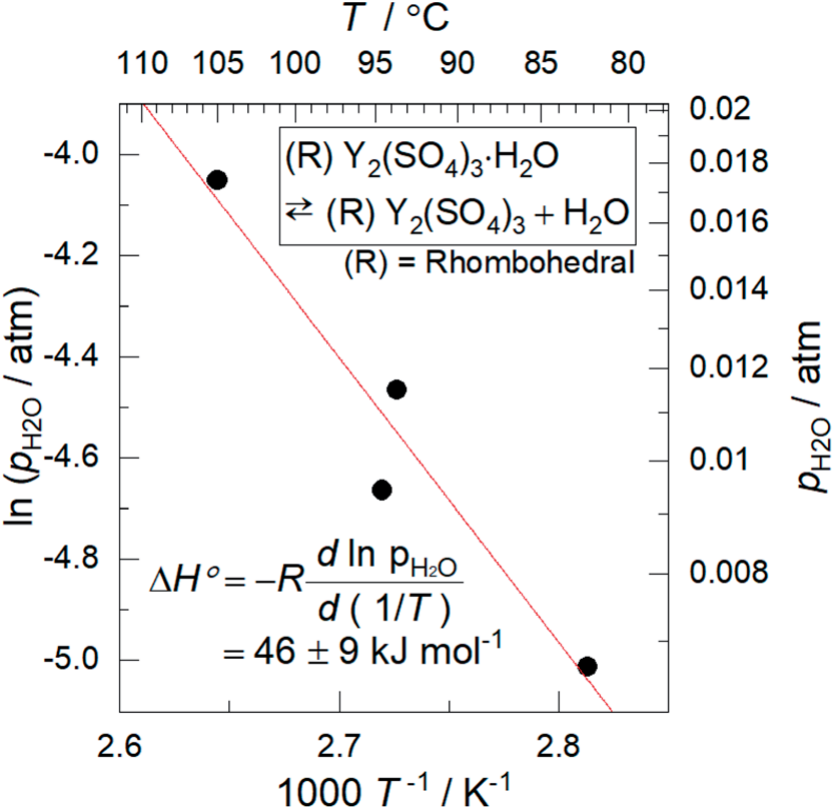
Van't Hoff plot for the high-temperature reaction of rhombohedral Y_2_(SO_4_)_3_·H_2_O. The equilibrium temperatures *T* are the estimated values from the TG measurements under four different *p*_H_2_O_ (ESI-2†). Because rhombohedral Y_2_(SO_4_)_3_·H_2_O dehydrates/hydrates *via* a two-phase reaction, the equilibrium temperature of the reaction (high-temperature reaction) should be a single temperature at each *p*_H_2_O_.

We also discuss in terms of heat storage density. The standard enthalpy change of the high-temperature dehydration reaction of rhombohedral Y_2_(SO_4_)_3_·H_2_O (reaction D) was estimated by a van't Hoff plot. The relationship between *p*_H_2_O_ and the equilibrium temperature of the reaction was assessed by TG measurements on rhombohedral Y_2_(SO_4_)_3_·H_2_O under four different *p*_H_2_O_ (ESI-2†). Fig. 11 shows the van't Hoff plot constructed from the equilibrium temperatures and *p*_H_2_O_. From the slope of the approximated straight line, the estimated standard enthalpy change Δ*H*° of rhombohedral Y_2_(SO_4_)_3_·H_2_O is 46 kJ (mol-H_2_O)^−1^ or 99 kJ (kg-hydrate)^−1^. The small gravimetric standard enthalpy change is attributed to the small hydration water content, *i.e.* the mass change with reaction D, equal to 3.72 mass%.

However, if the additional hydration reaction C with 10.3 mass% of mass change is utilized, a larger heat storage capacity is expected although the Δ*H*°/kJ (kg-hydrate)^−1^ of Y_2_(SO_4_)_3_·3H_2_O is not measured. Note that, among the dehydration/hydration reaction systems having similar equilibrium temperatures, the magnitude of Δ*H*°/kJ (kg-hydrate)^−1^ dominantly depends on the mass change. This is because the Δ*H*°/kJ (mol-H_2_O)^−1^ of dehydration reaction is approximately proportional to the equilibrium temperature.^27^ See (ESI-4)† for the relationship between the Δ*H*° and the equilibrium temperature. Thus, the mass change with the dehydration/hydration reaction is an indicator to simply evaluate the magnitude of the Δ*H*°/kJ (kg-hydrate)^−1^.

Here, the gravimetric energy densities of Y_2_(SO_4_)_3_·3H_2_O and Y_2_(SO_4_)_3_·H_2_O are compared with other materials. The Δ*H*° or mass change of Y_2_(SO_4_)_3_·3H_2_O (10.4 mass%) and Y_2_(SO_4_)_3_·H_2_O (99 kJ (kg-hydrate)^−1^, 3.72 mass%) is significantly lower than other salt hydrates with larger hydration numbers or with lower mass, for example, MgSO_4_·6H_2_O (1395 kJ (kg-hydrate)^−1^, 47.3 mass%),^5^ CaCl_2_·6H_2_O (1649 kJ (kg-hydrate)^−1^, 49.3 mass%),^28^ Na_2_S·5H_2_O (1784 kJ (kg-hydrate)^−1^, 53.6 mass%),^11^ and LiOH·H_2_O (1440 kJ (kg-hydrate)^−1^, 42.9 mass%).^20^ Compared with these salt hydrates, an advantage of Y_2_(SO_4_)_3_ system is the ease of handling since neither of aggregation due to the melting or deliquescence or skin formation covering the bulk particles has been confirmed in the hydration/dehydration reaction process. Therefore, Y_2_(SO_4_)_3_ system may be available for TCHS usage without developing composite materials with porous matrix and, in this respect, similar to CaSO_4_·0.5H_2_O and β-La_2_(SO_4_)_3_·H_2_O. The values of Δ*H*° and the mass change of Y_2_(SO_4_)_3_·3H_2_O or Y_2_(SO_4_)_3_·H_2_O are comparable to those of CaSO_4_·0.5H_2_O (240 kJ (kg-hydrate)^−1^ (ref. 26) and 6.21 mass%) and those of β-La_2_(SO_4_)_3_·H_2_O (156 kJ (kg-hydrate)^−1^ (ref. 15) and 3.08 mass%).

For TCHS application, heat output rate is also important but the performance of rhombohedral Y_2_(SO_4_)_3_ has never been evaluated yet. Regarding the hydration reaction rate shown in Fig. 3, the hydration reaction with hydration number change *x* of 1–3 completed within tens of minutes and that with *x* = ∼6 completed within 3 hours. These hydration reaction rates are comparable to those reported in the hydration of other salts, *i.e.* MgSO_4_,^5^ Na_2_S,^11^ β-La_2_(SO_4_)_3_,^17^ and CaCl_2_.^28^

## 5. Conclusion

We examine the hydration/dehydration of rhombohedral Y_2_(SO_4_)_3_ and their potential as a TCHS material by cyclic TG, high-temperature XRD, isothermal TG, isothermal hydration treatments, nitrogen gas adsorption, optical microscope observations, and TEM observations. TG measurements revealed that rhombohedral Y_2_(SO_4_)_3_ can hydrate/dehydrate reversibly below 130 °C with relatively small thermal hysteresis (less than 50 °C) and large change in hydration water content (above 10 mass%). Rhombohedral Y_2_(SO_4_)_3_ was found to be a favorable material in terms of the reaction temperature and the reaction behavior. The hydration reaction of rhombohedral Y_2_(SO_4_)_3_ proceeds *via* at least two steps: the high-temperature reaction at 80–130 °C and the low-temperature reaction at 30–100 °C in 0.02 atm of *p*_H_2_O_. The high-temperature hydration reaction proceeds by water insertion into rhombohedral Y_2_(SO_4_)_3_ to form rhombohedral Y_2_(SO_4_)_3_·H_2_O with only minor changes in the host crystal structure. In the low-temperature hydration reaction, rhombohedral Y_2_(SO_4_)_3_·H_2_O further hydrates to rhombohedral Y_2_(SO_4_)_3_·*x*H_2_O (*x* > 1) while maintaining the host structure. There is a possibility that rhombohedral Y_2_(SO_4_)_3_·*x*H_2_O has a nonstoichiometric hydration number *x*. At lower temperature (at most 27 °C in 0.02 atm of *p*_H_2_O_), it finally hydrates to Y_2_(SO_4_)_3_·8H_2_O which has a different crystal structure. Furthermore, another intermediate hydrate Y_2_(SO_4_)_3_·3H_2_O is formed in the hydration reaction under relatively high *p*_H_2_O_ (>0.08 atm) and intermediate temperature.

Regarding the application of rhombohedral Y_2_(SO_4_)_3_ to thermochemical heat pumps for energy upgrades, the hydration of rhombohedral Y_2_(SO_4_)_3_ to rhombohedral Y_2_(SO_4_)_3_·H_2_O can be used in the same manner as an existing candidate material CaSO_4_·0.5H_2_O in terms of reaction temperature and *p*_H_2_O_. However, the standard enthalpy change Δ*H*° of the dehydration of rhombohedral Y_2_(SO_4_)_3_·H_2_O (46 kJ mol^−1^) is not sufficient. The hydration of rhombohedral Y_2_(SO_4_)_3_·*x*H_2_O to Y_2_(SO_4_)_3_·3H_2_O (hydration water content: 11.6 mass% anhydrous basis) or Y_2_(SO_4_)_3_·8H_2_O (hydration water content: 30.9 mass% anhydrous basis) enables a larger change in the hydration number and thereby heat storage density, which is characteristic of this reaction system. Concerning the magnitude of the temperature lift on the practical use, the hydration to form Y_2_(SO_4_)_3_·3H_2_O may be available but that to form Y_2_(SO_4_)_3_·8H_2_O is not suitable since the temperature lift is as small as approximately 10 °C.

Finally, microstructure formed during the dehydration reaction from Y_2_(SO_4_)_3_·8H_2_O to rhombohedral Y_2_(SO_4_)_3_ was investigated. The estimated surface area of rhombohedral Y_2_(SO_4_)_3_ is 7 m^2^ g^−1^ while that of the original Y_2_(SO_4_)_3_·8H_2_O is significantly smaller (0.05 m^2^ g^−1^). Correspondingly, rhombohedral Y_2_(SO_4_)_3_ has a fine microstructure with crevices and voids at intervals of a-few-micrometers. Such a fine microstructure is formed by initial dehydration reaction from Y_2_(SO_4_)_3_·8H_2_O and expected to enhance the dehydration/hydration reaction rates. If the microstructure size can be further improved in the synthesis of rhombohedral Y_2_(SO_4_)_3_, a much faster hydration rate is expected.

## Conflicts of interest

There are no conflicts to declare.

## Supplementary Material

RA-010-D0RA02566F-s001
