# Supplementary material for: Multi-step hydration/dehydration mechanisms of rhombohedral Y2(SO4)3: a candidate material for low-temperature thermochemical heat storage
Source: RSC Adv. 2020 Apr 21;10(26):15604–13. doi: 10.1039/d0ra02566f (PMC9052420; doi:10.1039/d0ra02566f)
Supplement: RA-010-D0RA02566F-s001 [file RA-010-D0RA02566F-s001.pdf]

Electronic Supplementary Material (ESI) for RSC Advances.
This journal is © The Royal Society of Chemistry 2020

Supplemental Information

SI-1. Crystal structures of rare-earth sulfates

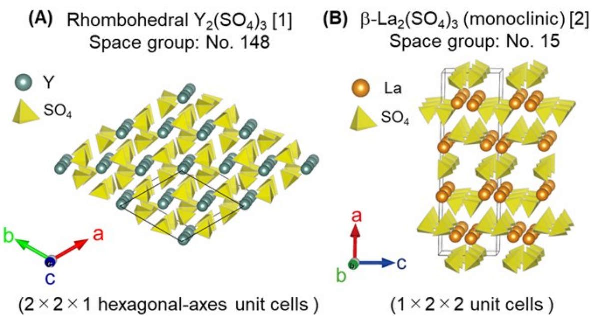

Figure S1. (A) Crystal structures of rhombohedral $Y_2(SO_4)_3$ [1] represented in hexagonal axial system for visibility. (B) Crystal structures of β-$La_2(SO_4)_3$. It has the same crystal structure as $Nd_2(SO_4)_3$ reported by Sirotinkin *et al.*.[2] Solid black line in each crystal structure show the unit cells of them.

References

[1] R. Perret, B. Rosso and J. Loriers, *Bull. Soc. Chim. Fr.* 1968, **7**, 2698–2699.

[2] S. P. Sirotinkin, V. A. Efremov, L. M. Kovba and A. N. Pokrovskii, *Sov. Phys. Crystallogr.* 1978, **22**, 725–726.

SI-2. TG measurements of rhombohedral $Y_2(SO_4)_3{\cdot}xH_2O$ ($0 \leq x \leq 1$) under four different p_{H_2O}

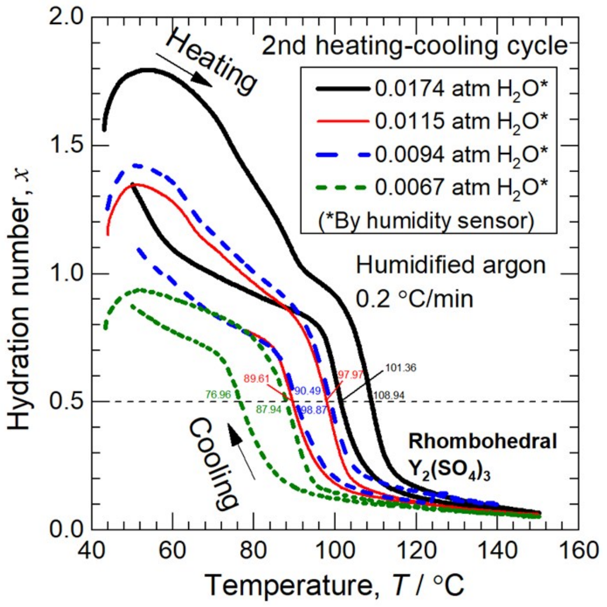

Figure S2. TG curves of rhombohedral $Y_2(SO_4)_3{\cdot}xH_2O$ ($0 \leq x \leq 1$) under four different p_{H_2O} (0.0174, 0.0115, 0.0094, and 0.0067 atm). These TG curves are collected during the 2nd heating-cooling cycle after the 1st cycle where the starting material $Y_2(SO_4)_3{\cdot}8H_2O$ is dehydrated to rhombohedral $Y_2(SO_4)_3$ in the heating step and rhombohedral $Y_2(SO_4)_3$ is hydrated to $Y_2(SO_4)_3{\cdot}xH_2O$ in the cooling step. Vertical axis represents hydration number x of $Y_2(SO_4)_3{\cdot}xH_2O$, which was calculated assuming the sample mass at 300 °C on the 1st heating as that of anhydrate. The average values of dehydration and hydration temperatures at which hydration number $x = 0.5$ were regarded as an approximate thermodynamic equilibrium temperature of the reaction. They are shown in Table S1.

Table S1. The values of hydration/dehydration temperatures read on TG curves and the estimated equilibrium temperatures with four different p_{H_2O}.

p_{H_2O} (atm)	Hydration temp. (°C)	Dehydration temp. (°C)	Equilibrium temp.* (°C)
0.0174	101.36	108.94	105.15
0.0115	89.61	97.97	93.79
0.0094	90.49	98.87	94.68
0.0067	76.96	87.94	82.45

* Equilibrium temperature is assumed to be the average of hydration and dehydration temperatures. Here, the estimated equilibrium temperatures have errors of approximately 10 °C at most because the high-temperature dehydration/hydration reactions exhibit a thermal hysteresis of approximately 20 °C on the TG curves.

SI-3. Estimated specific surface areas of rhombohedral $Y_2(SO_4)_3$ and $Y_2(SO_4)_3 \cdot 8H_2O$ based on BET method

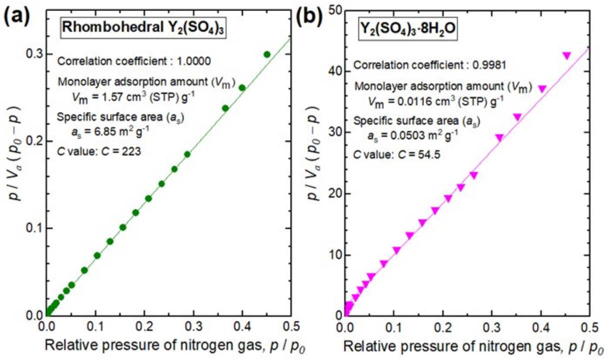

Figure S2. BET-plots of rhombohedral $Y_2(SO_4)_3$ (a) and $Y_2(SO_4)_3 \cdot 8H_2O$ (b) produced from the gas adsorption isotherms shown in Fig. 7. Approximate straight lines are drawn by a least-squares method using plots in the p/p_0 range from 0.05 to 0.30 where the BET equation holds. Correlation coefficients are calculated from the plots in the p/p_0 range from 0.05 to 0.30. (a) Monolayer adsorption amount: V_m = 1.57 cm^3 (STP) g^{-1}, Specific surface area: a_s = 6.85 m^2 g^{-1}, C value: C = 223. (b) Monolayer adsorption amount: V_m = 0.0116 cm^3 (STP) g^{-1}, Specific surface area: a_s = 0.0503 m^2 g^{-1}, C value: C = 54.5.

SI-4 Relations between the equilibrium temperature and the standard enthalpy change of dehydration reaction of various hydroxides and hydrates.

Considering the dehydration reaction of hydroxides or salt hydrates represented by the typical reaction formula below, the standard enthalpy change ΔH° / kJ (mol-H_2O)$^{-1}$ generally increases as the equilibrium temperature becomes higher.

$$\mathrm{M}\cdot n\mathrm{H_2O}\ (\text{solid}) \rightleftarrows \mathrm{M}\ (\text{solid}) + n\mathrm{H_2O}\ (\text{gas}) \quad (\text{M: oxides or anhydrous salts})$$

In the dehydration reaction, the ΔH° in arbitrary water vapor pressure $p_{\mathrm{H_2O}}$ and at the equilibrium temperature T^* is represented by the following equation using the standard entropy change ΔS° and gas constant R: $\Delta H^\circ = T^*\left(\Delta S^\circ + nR\ln p_{\mathrm{H_2O}}\right)$. Here, ΔS° can be approximated to the standard entropy of gaseous H_2O, $\Delta S^\circ \approx nS^\circ(\mathrm{H_2O})$, because the $nS^\circ(\mathrm{H_2O})$ is much higher than the difference of standard entropy between dehydrated phase M and hydrated phase M·nH_2O, $S^\circ(\mathrm{M}) - S^\circ(\mathrm{M}\cdot n\mathrm{H2O})$. Therefore, ΔH° is also approximated to the following equation: $\Delta H^\circ \approx nT^*\left(S^\circ(\mathrm{H_2O}) + R\ln p_{\mathrm{H_2O}}\right)$. Based on the approximation, the ΔH° is dominantly determined by the hydration number change n and the equilibrium temperature T^* and little depends on the solid phase species M. As an example, Table S1 shows the ΔH°, ΔS° and T^* of the dehydration reaction of eleven metal hydroxides and five salt hydrates with 1 mole of hydration number change n. Figure S1 shows the relationship between the ΔH° and the T^* of compounds shown in Table S1. It is confirmed that the ΔH° is approximately proportional to the T^*.

Thus, among the dehydration/hydration reaction systems having similar equilibrium temperatures, ΔH° approximately depends on only the hydration number change n, in other words, the mass change (mass%) with dehydration reaction.

Table S1. Standard enthalpy change, entropy change and equilibrium temperature of the dehydration reaction of eleven metal hydroxides and five salt hydrates.

Hydrate/Anhydrate	$\Delta H^{\circ}_{298\,K}$ / kJ (mol-H_2O)$^{-1}$	$\Delta S^{\circ}_{298\,K}$ / J (mol-H_2O)$^{-1}$ K^{-1}	T^* / °C
$Cu(OH)_2$ / CuO [1]	45.20	144.5	39.57
$Fe(OH)_2$ / FeO [1]	55.13	161.7	67.75
$Ni(OH)_2$ / NiO [2]	48.17	138.5	74.67
$Zn(OH)_2$ / ZnO [2]	53.14	150.9	79.01
$Sn(OH)_2$ / SnO [2]	33.47	91.88	91.17
$Be(OH)_2$ (β) / BeO [1]	55.66	152.5	91.75
$Co(OH)_2$ / CoO [1]	59.93	162.9	94.66
$Mg(OH)_2$ / MgO [1]	81.60	152.7	261.3
$Ca(OH)_2$ / CaO [1]	109.2	143.6	486.8
$Sr(OH)_2$ / SrO [1]	135.0	147.4	642.8
$Ba(OH)_2$ / BaO [1]	150.9	152.3	718.1
$CdSO_4{\cdot}H_2O$ / $CdSO_4$ [2]	64.44	157.8	135.2
$CuSO_4{\cdot}H_2O$ / $CuSO_4$ [1]	72.65	152.0	204.9
$ZnSO_4{\cdot}H_2O$ / $ZnSO_4$ [1]	76.89	153.9	226.3
$CdCl_2{\cdot}H_2O$ / $CdCl_2$ [2]	55.11	136.5	130.7
$MnCl_2{\cdot}H_2O$ / $MnCl_2$ [2]	65.89	130.5	231.7

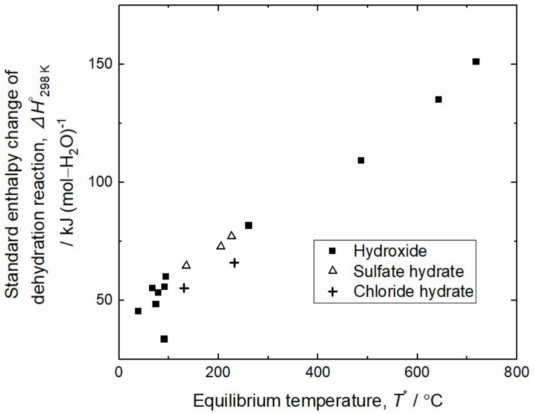

Figure S1. Relationship between ΔH° and $T*$ of dehydration reaction of eleven metal hydroxides and five salt hydrates.

References

[1] I. Barin, Thermodynamic data of pure substances, VCH, 1993.

[2] D. Wagman, The NBS table of chemical thermodynamic properties, *J Phys Chem Ref Data*, 1982, **86**, 11–20.
